# Optimizing the Aspect Ratio of Nanopatterned Mesoporous TiO_2_ Thin-Film Layer to Improve Energy Conversion Efficiency of Perovskite Solar Cells

**DOI:** 10.3390/ijms222212235

**Published:** 2021-11-12

**Authors:** Hwa-Young Yang, Ana Chuquer, Seung-Hee Han, Gangasagar Sharma Gaudel, Xuan-Hung Pham, Hyung-Mo Kim, Won-Ju Yun, Bong-Hyun Jun, Won-Yeop Rho

**Affiliations:** 1 School of Energy and Chemical Engineering, Ulsan National Institute of Science and Technology (UNIST), 50 UNIST-gil, Eonyang-eup, Ulju-gun, Ulsan 44919, Korea; hyang@unist.ac.kr; 2 School of Bioenvironmental Chemistry, Jeonbuk National University, 567 Baekje-daero, Deokjin-gu, Jeonju-si 54896, Jeollabuk-do, Korea; chuquerana@gmail.com; 3 School of International Engineering and Science, Jeonbuk National University, 567 Baekje-daero, Deokjin-gu, Jeonju-si 54896, Jeollabuk-do, Korea; rinmin0616@naver.com; 4 Graduate School of Integrated Energy-AI, Jeonbuk National University, 567 Baekje-daero, Deokjin-gu, Jeonju-si 54896, Jeollabuk-do, Korea; gangasagarsg@gmail.com; 5 Department of Bioscience and Biotechnology, Konkuk University, 120 Neungdong-ro, Gwangjin-gu, Seoul 05029, Korea; phamricky@gmail.com (X.-H.P.); hmkim0109@konkuk.ac.kr (H.-M.K.); 6 Department of Physics, Jeonbuk National University, 567 Baekje-daero, Deokjin-gu, Jeonju-si 54896, Jeollabuk-do, Korea; wjyun@jbnu.ac.kr

**Keywords:** perovskite solar cell, nanopattern, aspect ratio, transmittance

## Abstract

The energy conversion efficiency (ECE) (η), current density (*J_sc_*), open-circuit voltage (*V_oc_*), and fill factor (*ff*) of perovskite solar cells were studied by using the transmittance of a nanopatterned mesoporous TiO_2_ (mp-TiO_2_) thin-film layer. To improve the ECE of perovskite solar cells, a mp-TiO_2_ thin-film layer was prepared to be used as an electron transport layer (ETL) via the nanoimprinting method for nanopatterning, which was controlled by the aspect ratio. The nanopatterned mp-TiO_2_ thin-film layer had a uniform and well-designed structure, and the diameter of nanopatterning was 280 nm. The aspect ratio was controlled at the depths of 75, 97, 127, and 167 nm, and the perovskite solar cell was fabricated with different depths. The ECE of the perovskite solar cells with the nanopatterned mp-TiO_2_ thin-film layer was 14.50%, 15.30%, 15.83%, or 14.24%, which is higher than that of a non-nanopatterned mp-TiO_2_ thin-film layer (14.07%). The enhancement of ECE was attributed to the transmittance of the nanopatterned mp-TiO_2_ thin-film layer that is due to the improvement of the electron generation. As a result, better electron generation affected the electron density, and *J_sc_* increased the *V_oc_*, and *ff* of perovskite solar cells.

## 1. Introduction

Renewable energy is a sustainable method for generating affordable electricity with a lower carbon footprint compared with other conventional resources [1]. Owing to environmental problems, the solar cell industry has developed rapidly in recent years. Therefore, many countries have invested in projects in order to discover better materials for solar cells [2,3]. Solar cells are devices that transform sunlight into electricity, and they have a very low environmental impact [2,4]. They have been a topic of interest in the green energy field, owing to the possibility of solar cells becoming an alternative energy source that can replace conventional energy sources [5]. Previous studies have aimed to improve the efficiency of solar cells [2,5,6]. These studies have achieved important improvements, and the dominance of silicon solar cells has been established in the market, exhibiting an energy conversion efficiency (ECE) higher than 20% [2]. However, as perovskite solar cells can achieve better ECE, they are much more convenient than other solar cells [7,8,9]. They have various advantages, such as higher efficiencies, stability, and a lower cost of production [10,11]. Perovskite is a semiconductor material that was discovered by Gustav Rose in 1839, and it was named after the mineralogist L. A. Perovski [12]. Perovskites possess a tunable chemical structure ABX_3_, where “A” and “B” are cations and “X” is usually an oxide or halide. In 2009, the scientist Miyasaka reported the first incorporation of perovskite in solar cells, with just 3.8% ECE [13]. The performance and stability of their perovskite-based solar cell was low, the wavelength range of absorption was wide and the open-circuit voltages (*V_oc_*) were higher [13]. The conventional perovskite solar cell structure comprises a transparent conduction oxide such as fluorine-doped tin oxide (FTO) or indium tin oxide (ITO), an electron transport layer (ETL), a perovskite, a hole transport layer (HTL), and an electrode [14,15]. Over some years, many studies have investigated these solar cells and obtained important results. Lee et al. introduced a solid-state electrolyte/hole transport material instead of liquid electrolytes [16]. In 2012, Grätzel and Park reported the usage of a perovskite material as a sensitizer in solid type dye-sensitized solar cells (DSSCs) [17]. In the same year, Kanatzid introduced all-solid-state dye-sensitized solar cells with perovskites such as CsSnI_3_ as the *p*-type inorganic semiconductor instead of the electrolyte [18]. In addition, Snaith discovered the meso-superstructured solar cells. In meso-superstructured solar cells, the mesoporous *n*-type TiO_2_ is replaced by insulating Al_2_O_3_ and used at the bottom of the electrode [16]. The planar structure was introduced in 2013, in which a perovskite layer was inserted between the ETL and HTL [19]. The pillared structure of perovskite solar cells is characterized by using mesoporous TiO_2_ (mp-TiO_2_) as an ETL [20,21]. The best ECE achieved for perovskite solar cells was 25.5%, recorded by the Ulsan National Institute of Science and Technology (UNIST) in South Korea [22,23,24]. Some studies have introduced new strategies and methods associated with ETL, such as a 2D photonic crystal array that enhances light harvesting and reduces the contact resistance with the perovskite layer [25]. A grating pattern made by diffraction grating into the TiO_2_ scaffold and antireflection layer improves light transmittance and consequently light absorption [26]. A constructed diffraction grating has been introduced into the perovskite-active layer by commercial optical discs (CD-R and DVD-R) to reduce recombination [27]. Other studies have highlighted interesting properties of mp-TiO_2_, such as stability, high transparency, and a suitable band gap for perovskite solar cells [28,29,30,31]. The mp-TiO_2_ facilitates the transport of the electrons and the absorption of light [32,33]. In terms of ECE, mp-TiO_2_ was used to minimize recombination losses by reducing the interface defects between the perovskite and the FTO substrate [34]. Consecutively, a mp-TiO_2_ thin-film layer has been commonly used as an ETL in perovskite solar cells owing to its higher uniformity and well-developed structure [24,35,36,37]. However, a mp-TiO_2_ thin-film exhibited some ECE losses caused by poor light harvesting [38,39]. Light harvesting is a crucial factor for the improvement of the ECE in solar cells [40,41,42]. In the literature, there have been numerous strategies to improve light harvesting, such as a light-scattering layer [43], anti-reflection outer surfaces [44,45], 3-D nanostructures [46], and nanostructured plasmonic contacts [47]. A nanopatterned mp-TiO_2_ thin-film was introduced by using a new method to improve light harvesting in perovskite solar cells [7,48,49,50]. Even though the use of only nanopatterned mp-TiO_2_ results in the improvement of ECE values, a better method is required to enhance light harvesting. In this paper, we successfully report the control of the aspect ratio of a nanopatterned mp-TiO_2_ thin-film with uniform, clear, and well-designed structures to improve light harvesting in perovskite solar cells. At the beginning of this paper, we describe the fabrication of perovskite solar cells using the imprinting method of a perfluoropolyether (PFPE) mold. Furthermore, there are comparisons and an analysis of the photovoltaic properties of the mp-TiO_2_ thin-film layer with different nanopatterning depths. Finally, we conclude by providing comments on the results and our expectations for future investigations.

## 2. Results and Discussion

### 2.1. Overall Scheme of the Process for Fabricating Perovskite Solar Cells with a Nanopatterned Mesoporous TiO_2_ (mp-TiO_2_) Thin-Film Layer

**Figure 1:**
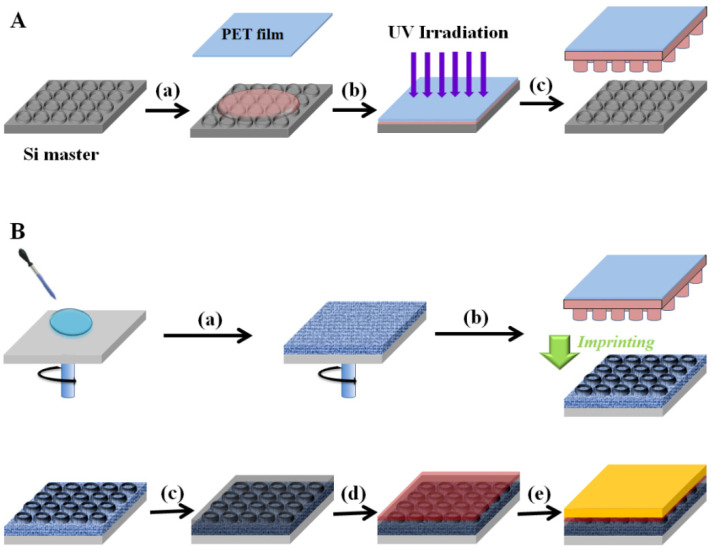
(**A**) Preparation of perfluoropolyether (PFPE) mold from the Si master: (**a**) PFPE resin and polyethylene terephthalate (PET) film, (**b**) irradiation of UV, and (**c**) PFPE mold. (**B**) Fabrication of perovskite solar cells with a nanopatterned mesoporous TiO_2_ (mp-TiO_2_) thin-film layer: (**a**) coating of mp-TiO_2_ thin-film layer, (**b**) nanopatterning of mp-TiO_2_ thin-film layer through imprinting method, (**c**) coating of perovskite, (**d**) coating of spiro-OMeTAD as hole transport material, and (**e**) evaporation of Au.

Figure 1 shows the overall scheme of the fabrication process of perovskite solar cells with a nanopatterned mesoporous TiO_2_ (mp-TiO_2_) thin-film layer. Figure 1A shows the preparation of the perfluoropolyether (PFPE) mold from a Si master. The PFPE resin was coated on the Si master and covered with a polyethylene terephthalate (PET) film as a flexible backplane, as shown in Figure 1A(a). After UV irradiation, the PFPE resin underwent polymerization for hardening, as shown in Figure 1A(b). The PET film contained the PFPE mold, which was obtained from the PFPE resin using polymerization and was separated from the Si master, as shown in Figure 1A(c). Figure 1B shows the fabrication of perovskite solar cells with a nanopatterned mp-TiO_2_ thin-film layer. The mp-TiO_2_ thin-film layer was prepared on a fluorine-doped tin oxide (FTO) glass from a solution of TiO_2_ nanoparticles (NPs) by using a spin coater, as shown in Figure 1B(a). It was then imprinted by using the PFPE mold for nanopatterning on the mp-TiO_2_ thin-film layer as shown in Figure 1B(b). To imprint the mp-TiO_2_ thin-film layer using the PFPE mold, the mp-TiO_2_ thin-film layer was pre-baked at 70 °C for 1 min to evaporate the solvent. After imprinting, the mp-TiO_2_ thin-film layer was sintered. The perovskite and hole transport materials, such as spiro-OMeTAD, were coated on the nanopatterned mp-TiO_2_ thin-film layer, as shown in Figure 1B(c,d). Au was evaporated by using a metal evaporator, as shown in Figure 1B(e). The device structure was FTO/nanopatterned mp-TiO_2_/perovskite/sprio-OMeTAD/Au.

### 2.2. FE-SEM, Optical, and FIB Images of Perovskite Solar Cells with a Nanopatterned Mesoporous TiO_2_ (mp-TiO_2_) Thin-Film Layer

**Figure 2:**
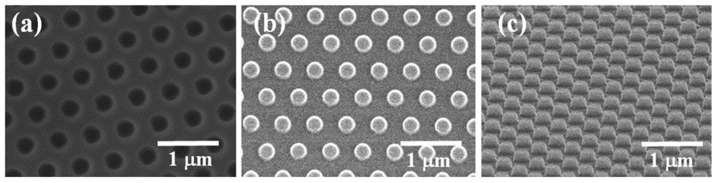
FE-SEM images of (**a**) Si master, (**b**) PFPE mold, and (**c**) tilted view of PFPE mold.

Figure 2 shows the field emission scanning electron microscopy (FE-SEM) images of the patterned Si master, PFPE mold, and tilted view of PFPE mold. The PFPE resin was mixed with a UV-curable resin, and the mixture was dropped on the Si master. The PET film covered the mixture as a flexible backbone. After the PET film was rolled and irradiated by UV, the PFPE mold was prepared. The pore size was 250 nm, and the interpore distance was 250 nm. The PFPE mold on the PET film was used for nanopatterning the mp-TiO_2_ thin-film layer via the nanoimprinting method.

**Figure 3:**
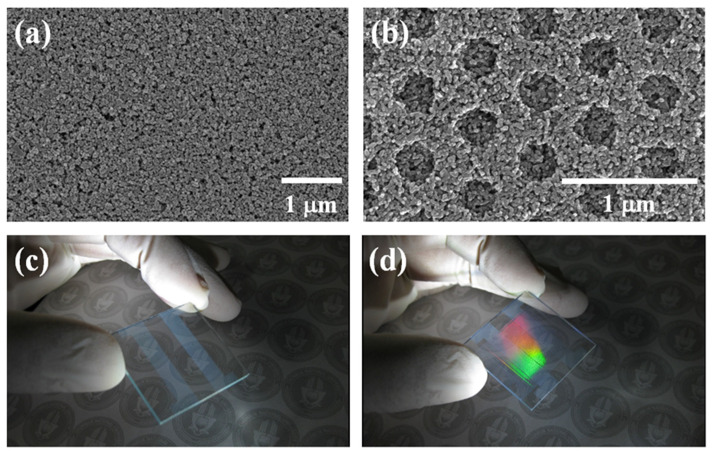
Images of nanopatterned mp-TiO_2_ thin-film layer. (**a**) Non-nanopatterned mp-TiO_2_ thin-film layer, and (**b**) nanopatterned mp-TiO_2_ thin-film layer using FE-SEM. (**c**) Non-nanopatterned mp-TiO_2_ thin-film layer, and (**d**) nanopatterned mp-TiO_2_ thin-film layer using optical imaging.

Figure 3 shows the FE-SEM and optical images of non-nanopatterned and nanopatterned mp-TiO_2_ thin-film layers. The non-nanopatterned mp-TiO_2_ thin-film layer shows only TiO_2_ NPs (Figure 3a). The nanopatterned mp-TiO_2_ thin-film layer shows uniform and well-designed pores on the surface, such as the moth-eye nanostructure, which was formed using the nanoimprinting method. The pore size was 280 nm, and the interpore distance was 220 nm, as shown in Figure 3b. Figure 3c,d shows optical images under the light. The non-nanopatterned mp-TiO_2_ thin-film layer shows the pattern of FTO under the light (Figure 3c). However, the nanopatterned mp-TiO_2_ thin-film layer displays rainbow colors under the light (Figure 3d), owing to the diffraction grating created by the nanopatterned mp-TiO_2_ thin-film layer.

**Figure 4:**
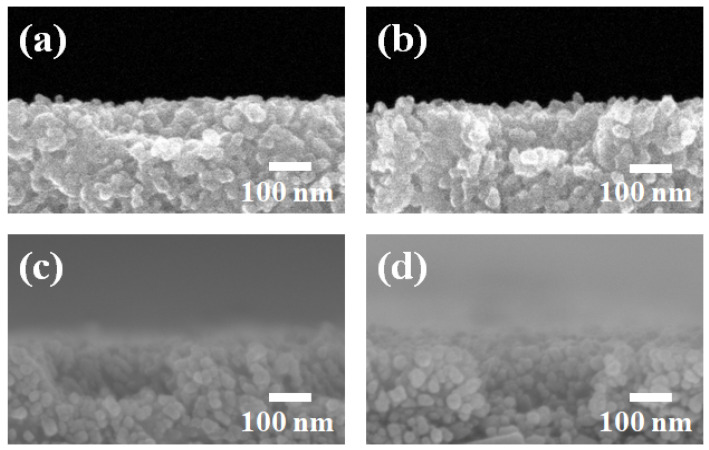
FE-SEM images of nanopatterned mp-TiO_2_ thin-film layer. Mp-TiO_2_ thin-film layer nanopatterned at the depths of (**a**) 75 nm, (**b**) 97 nm, (**c**) 127 nm, and (**d**) 167 nm.

Figure 4 shows the FE-SEM images of the mp-TiO_2_ thin-film layer for different nanopatterning depths. The diameter of the pore was 280 nm, and the depths of the pore were 75, 97, 127, and 167 nm. The aspect ratio defined by the depth and diameter of the nanopatterned mp-TiO_2_ thin-film layer was controlled by different depths. Perovskite solar cells were fabricated with the nanopatterned mp-TiO_2_ thin-film layer at different depths to optimize the transmittance by using the aspect ratio.

**Figure 5:**
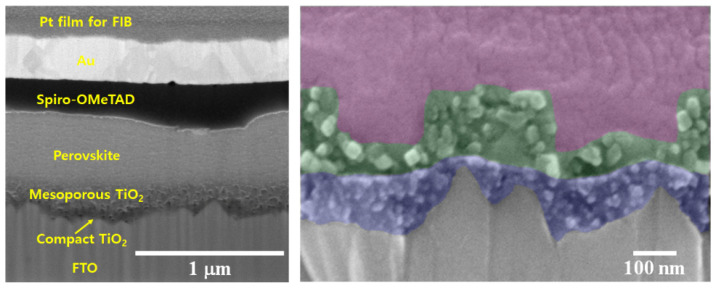
Cross-sectional images of perovskite solar cells using focused ion beam (FIB) and FE-SEM image with color-enhanced layer boundaries.

Figure 5 shows the cross-sectional images of perovskite solar cells suing focused ion beam (FIB) and FE-SEM image with color-enhanced layer boundaries. The thickness of the mp-TiO_2_ thin-film layer, perovskite, Spiro-OMeTAD, and Au are 185 nm, 130 nm, 480 nm, 247 nm, and 262 nm, respectively. The cross-sectional image of perovskite solar cells with mp-TiO_2_ thin-film layer shows the compact layer (blue), mp-TiO_2_ thin-film layer (green), and perovskite layer (purple).

### 2.3. XRD Images of Perovskite Solar Cells with a Nanopatterned Mesoporous TiO_2_ (mp-TiO_2_) Thin-Film Layer

**Figure 6:**
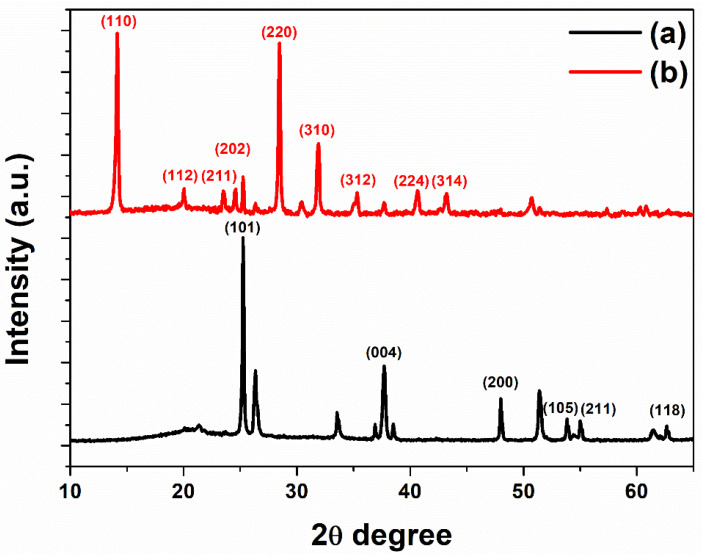
XRD images of (**a**) nanopatterned mp-TiO_2_ thin-film layer, and (**b**) perovskite on nanopatterned mp-TiO_2_ thin-film layer.

The crystallinity values of the nanopatterned mp-TiO_2_ thin-film layer and perovskite were confirmed by X-ray diffraction (XRD) patterns as shown in Figure 6. The nanopatterned mp-TiO_2_ thin-film layer shows the anatase phases from (101), (004), (200), (105), (211), and (118) peaks at 2θ of 25.2°, 38°, 48°, 54°, 55.5°, and 62.5°. The perovskite shows the cubic phases from (110), (112), (211), (202), (220), (310), (312), (224), and (314) peaks at 2θ of 14.15°, 20.05°, 23.5°, 24.6°, 28.4°, 31.9°, 35.3°, 40.6°, and 43.2°. The high intense peaks at 14.1° and 28.4° indicate a crystalline perovskite. The XRD results show that the crystalline perovskite was well formed on the nanopatterned mp-TiO_2_ thin-film layer; thus it could achieve better energy conversion efficiency in a perovskite solar cell.

### 2.4. Transmittance Data of Perovskite Solar Cells with a Nanopatterned Mesoporous TiO_2_ (mp-TiO_2_) Thin-Film Layer

**Figure 7:**
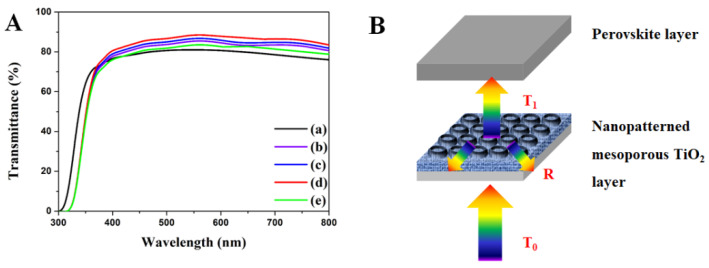
(**A**) Transmittance of nanopatterned mp-TiO_2_ thin-film layer. (**B**) Schematic of the transmittance and reflectance of nanopatterned mp-TiO_2_ thin-film layer. (**a**) Non-nanopatterned mp-TiO_2_ thin-film layer. mp-TiO_2_ thin-film layer nanopatterned at the depths of (**b**) 75 nm, (**c**) 97 nm, (**d**) 127 nm, and (**e**) 167 nm.

The transmittance of the nanopatterned mp-TiO_2_ thin-film layer with different aspect ratios of diameter 280 nm and depths 75 nm, 97 nm, 127 nm, and 167 nm were measured using UV-vis spectra, as shown in Figure 7A. The schematic function of the transmittance and reflectance of the nanopatterned mp-TiO_2_ thin-film layer is shown in Figure 7B. The transmittance of the non-nanopatterned mp-TiO_2_ thin-film layer is higher than that of the nanopatterned mp-TiO_2_ thin-film layer at a wavelength of 380 nm. However, the transmittance of the non-nanopatterned mp-TiO_2_ thin-film layer is lower than that of the nanopatterned mp-TiO_2_ thin-film layer for wavelengths higher than 380 nm. The nanopatterned mp-TiO_2_ thin-film layer increases surface roughness and changes optical effects. Furthermore, it induces changes in the refractive index that affect the transmittance of light through a medium. The decrease in the refractive index increases the transmittance and vice-versa. For 75 nm, 97 nm and 127 nm depths, the transmittance increased, but it decreased at 167 nm depth [51]. This result may suggest that the refractive index of the nanopatterned depths of 75 nm, 97 nm, and 127 nm decreases but increases at 167 nm. This result shows that the aspect ratio of the nanopatterned structure affects the transmittance. In addition, if the angle of the reflected/diffracted light is greater than the critical angle, it causes a total internal reflection due to a decrease in the value of the refractive index of the TiO_2_ layer to air (refractive index of TiO_2_ (≈2.1), FTO (≈1.9), Glass (≈1.5), Air (=1.0)). The light is then reflected towards the mp-TiO_2_, and thus the transmittance to the perovskite layers increases [52]. Considering the perovskite materials, whose absorbance wavelength varies from 300 nm to 800 nm, the increment in transmittance of the nanopatterned mp-TiO_2_ thin-film layer affects the *J_sc_*, *V_oc_*, *ff*, and the ECE of perovskite solar cells.

### 2.5. Characterization of Perovskite Solar Cells with and without the Nanopatterned Mesoporous TiO_2_ (mp-TiO_2_) Thin-Film Layer

**Figure 8:**
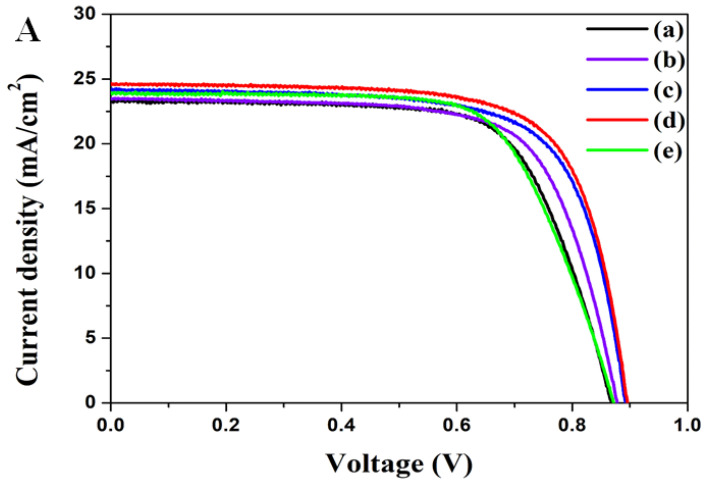

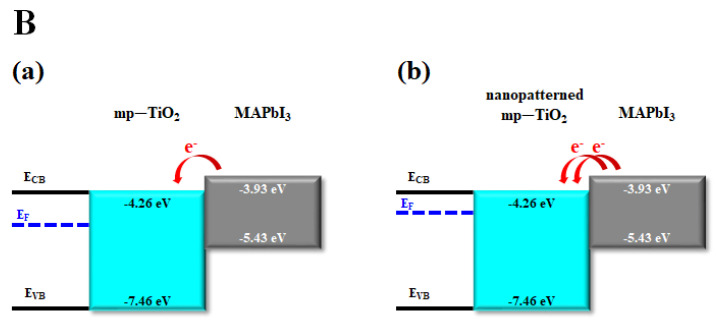
(**A**) shows the I-V characteristics of perovskite solar cells with and without a nanopatterned mp-TiO_2_ thin-film layer. A shows (**a**) non-nanopatterned perovskite solar cells and perovskite solar cells with a nanopatterned mp-TiO_2_ thin-film layer with the depths of (**b**) 75 nm, (**c**) 97 nm, (**d**) 127 nm, and (**e**) 167 nm. (**B**) shows the electron equilibration of the apparent Fermi level on the nanopatterned mp-TiO_2_ thin-film layer. (**a**) denotes the non-nanopatterned mp-TiO_2_ thin-film layer, and (**b**) denotes the nanopatterned mp-TiO_2_ thin-film layer.

The I-V characteristics of perovskite solar cells were characterized under one condition of the sun, as shown in Figure 8A. The results of current density (*J_sc_*), open-circuit voltage (*V_oc_*), fill factor (*ff*), and ECE (η) are summarized in Table 1. The ECE of perovskite solar cells without the nanopatterned mp-TiO_2_ thin-film layer is 14.07%, while for perovskite solar cells with the nanopatterned mp-TiO_2_ thin-film layer, it increased from 14.50% to 15.83%, with an increase in depth from 75 nm to 127 nm. This is due to an increment in *J_sc_* from 23.50 mA/cm^2^ to 24.62 mA/cm^2^, as the nanopatterned mp-TiO_2_ thin-film layer leads to a better transmittance that enhances the electron generation. *V_oc_* increases with an increase of *J_sc_*. This is because, as the electron generation increases, as shown in Figure 8B, the electron density and Fermi level of the nanopatterned mp-TiO_2_ thin-film layer increase. *V_oc_* is determined by the conduction band of the nanopatterned mp-TiO_2_ thin-film layer and the valence band of perovskite. Thus, *V_oc_* also increases from 0.869 V to 0.896 V.

**Figure 9:**
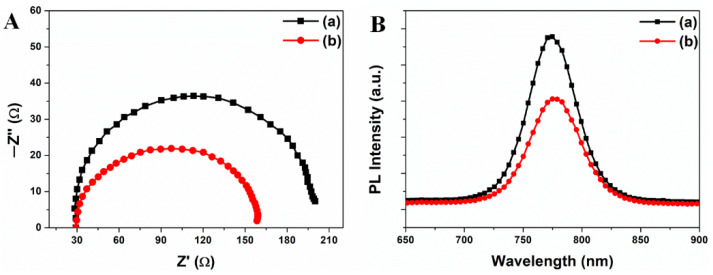
(**A**) shows the electrochemical impedance spectroscopy of perovskite solar cells with a non-nanopatterned (**a**) and nanopatterned (**b**) TiO_2_ thin-film layer. (**B**) shows the photoluminescence (PL) spectra of perovskite solar cells with a non-nanopatterned (**a**) and nanopatterned (**b**) TiO_2_ thin-film layer.

To confirm the results, the perovskite solar cells with and without a nanopatterned mp-TiO_2_ thin-film layer were characterized by the electrochemical impedance spectroscopy (EIS) and photoluminescence (PL) spectroscopy, as shown in Figure 9A,B. In electrochemical impedance spectroscopy, the recombination resistance (*R_rec_*) of perovskite solar cells with the nanopatterned mp-TiO_2_ thin-film layer was suppressed compared to those without the nano-patterned mp-TiO_2_ thin-film layer. The decrement in recombination was affected by the increment in electron density and it was also affected the increment in *V_oc_*. Thus, the *V_oc_* of perovskite solar cells with nanopatterned mp-TiO_2_ thin-film layer increased. Moreover, in PL spectra, the perovskite materials with the nanopatterned mp-TiO_2_ thin-film layer quenched more compared to those without the nanopatterned mp-TiO_2_ thin-film layer. The generated electrons from perovskite solar cells with nanopatterned mp-TiO_2_ thin-film layer quenched more, which means that the generated electrons were transferred from perovskite to the nanopatterned mp-TiO_2_ thin-film layer effectively before recombination. It means that the transferred electrons increase the electron density of the nanopatterned mp-TiO_2_ thin-film layer. There is also a change in the Fermi level of the nanopatterned mp-TiO_2_ thin-film layer due to its conduction band. Thus, the *V_oc_* changed because *V_oc_* is determined by the conduction band of TiO_2_ and the valence band of perovskite materials. However, the ECE (*η*) of perovskite solar cells with a 167 nm depth nanopatterned mp-TiO_2_ thin-film layer decreased to 14.24% with a decrease in *V_oc_* and *ff*. From these results, the aspect ratio of nanopattern between the diameter and depth should be optimized for better ECE of perovskite solar cells.

**Figure 10:**
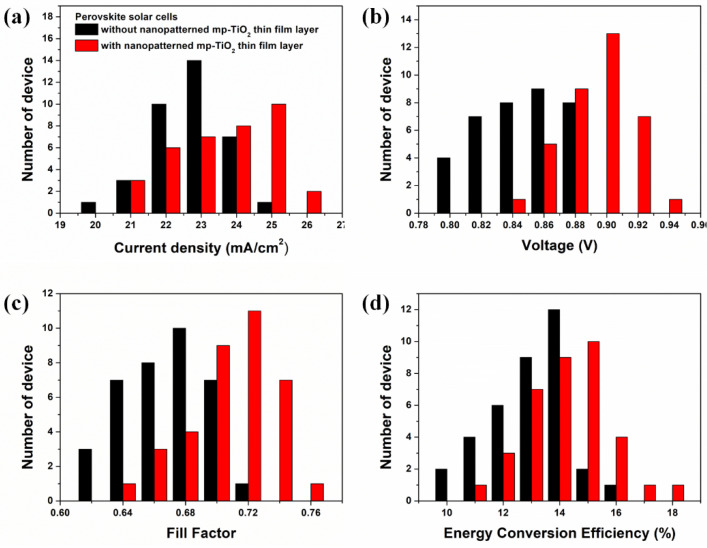
Histograms of perovskite solar cells without a nanopatterned (black) and with a nanopatterned (red) TiO_2_ thin-film layer. (**a**) is current density, (**b**) is voltage, (**c**) is fill factor, and (**d**) is energy conversion efficiency.

To demonstrate the repeatability of perovskite solar cells with and without a nanopatterned mp-TiO_2_ thin-film layer, the 36 perovskite solar cells with and without a nanopatterned mp-TiO_2_ thin-film layer were measured, and the data are illustrated in the histograms as shown in Figure 10.

The pattern of the IPCE spectra of perovskite solar cells is similar to the transmittance of a nanopatterned mp-TiO_2_ thin-film layer because transmittance is affected by *J_sc_*, *V_oc_*, *ff*, and the ECE of perovskite solar cells. The intensity of the IPCE spectra on perovskite solar cells with the non-nanopatterned mp-TiO_2_ thin-film layer is higher than that with the nanopatterned mp-TiO_2_ thin-film layer at a wavelength of 430 nm. However, the intensity of the IPCE spectra on perovskite solar cells with the non-nanopatterned mp-TiO_2_ thin-film layer is lower than that with the nanopatterned mp-TiO_2_ thin-film layer at wavelengths ranging from 430 nm to 800 nm. Higher intensity of the IPCE spectra indicates that more electrons are generated from 430 nm to 800 nm. More electron generation improves the electron density on the mp-TiO_2_ thin-film layer, which, in turn, increases *J_sc_*, *ff*, and *V_oc_*. Thus, the ECE of perovskite solar cells with the nanopatterned mp-TiO_2_ thin-film layer increases. The integrated *J_sc_* can be determined from Equation (1).

(1) $$J_{sc,\;int}=\frac{F\;\times \;\int \left(E_{e\lambda }\;\times \;IPCE\right)d\lambda }{N_{A}}$$

**Figure 11:**
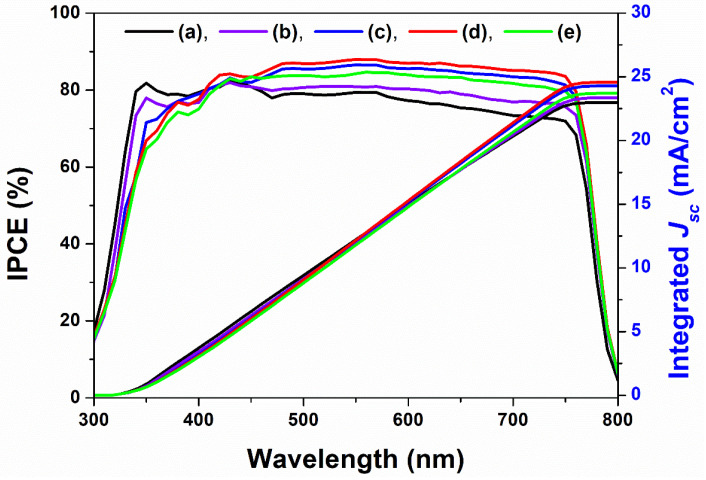
IPCE (incident photon to electron conversion efficiency) spectra, calculated by integrated *J_sc_* of perovskite solar cells with a non-nanopatterned and nanopatterned mp-TiO_2_ thin-film layer. (**a**) denotes perovskite solar cells with the non-nanopatterned mp-TiO_2_ thin-film layer and (**b**–**e**) denote perovskite solar cells with the nanopatterned mp-TiO_2_ thin-film layer with a depth of 75 nm, 97 nm, 127 nm, and 167 nm, respectively.

Here, *F* is the Faraday constant, *E_eλ_* is the solar spectral irradiance, and *N_A_* is the Avogadro constant. *J_sc_* is calculated by Equation (1), as shown in Figure 11. The integrated *J_sc_* is matched with the measured *J_sc_* of perovskite solar cells with the non-nanopatterned or nanopatterned mp-TiO_2_ thin-film layer.

## 3. Materials and Methods

### 3.1. Preparation of Perfluoropolyether (PFPE) Mold

The UV-curable resin was prepared by mixing the PFPE resin (Fluorolink MD700, Solvay Solexis, Milan, Italy) with 3% *w*/*w* photoinitiator (Darocur 1173, Sigma-Aldrich, St. Louis, MO, USA). The UV-curable resin was dropped on the patterned silicon substrate (i.e., Si master) and covered with polyethylene terephthalate (PET) film as a backplane. To spread the UV-curable resin between the PET film and Si master, the PET film was rolled over the resin and cross-linked by UV irradiation at 365 nm for 5 min. After curing, the PFPE mold was detached from the Si master.

### 3.2. Preparation of Nanopatterned mp-TiO_2_ Thin-Film Layer

The fluorine-doped tin oxide (FTO) glass was washed with water, ethanol, and acetone. The compact TiO_2_ precursor, which comprises 1 mL of titanium diisopropoxide bis(acetylacetonate) (75 wt% in isopropanol, Sigma-Aldrich, St. Louis, MO, USA) in 6 mL of butanol (99%, Daejung Chemicals, Siheung-si, Korea) was spin-coated on FTO at 4000 rpm for 20 s, followed by annealing at 250 °C for 10 min. This process was repeated twice. To form the mesoporous TiO_2_ (mp-TiO_2_) layer, 1 g of TiO_2_ paste (Ti-Nanoxide T/SP, Solaronix, Aubonne, Switzerland) was diluted in 12 mL of anhydrous ethanol (99.9%, Daejung Chemicals, Siheung-si, Korea). The dispersion solution was spin-coated on a compact TiO_2_/FTO. After prebaking on a hotplate at 70 °C for 1 min to evaporate the solvent slightly, the PFPE mold was placed on the mp-TiO_2_/compact TiO_2_/FTO substrate. The substrate was heated at 70 °C, and then a pressure of 2 bar was applied for 5 min. After removing the mold, the nanopatterned mp-TiO_2_/compact TiO_2_/FTO substrate was annealed at 500 °C for 1 h.

### 3.3. Preparation of Perovskite Precursor Solution

Methylammonium iodide (MAI) was prepared with 25 mL of methylamine (33 wt% in ethanol, Sigma-Aldrich, St. Louis, MO, USA) and 10 mL of hydroiodic acid (55~58%, Kanto Chemical, Tokyo, Japan) in a round-bottom flask with an ice bath for 2 h. The products were recovered using a rotary evaporator at 60 °C for 1 h. After evaporating, the precipitates were recrystallized with ethanol (99.9%, Daejung Chemicals, Siheung-si, Korea) and diethyl ether (99%, Daejung Chemicals, Siheung-si, Korea) several times and dried at 60 °C in a vacuum oven overnight. The perovskite precursor was prepared by stirring MAI and lead (II) chloride (99.999%, Sigma-Aldrich, St. Louis, MO, USA) at a 3:1 molar ratio, 45 wt% in *N,N*-dimethylformamide (99.8%, Sigma-Aldrich, St. Louis, MO, USA) at 90 °C for 1 h.

### 3.4. Fabrication of Perovskite Solar Cells

The perovskite precursor solutions were spin-coated on the nanopatterned mp-TiO_2_ layer by using the hot-casting technique at 90 °C and then annealed at 130 °C for 1 h. The hole transport materials were prepared by stirring 73.5 mg of spiro-OMeTAD (99.62%, Feiming Chemical Limited, Shenzhen, China), 17 µL of Bis (trifluoromethane)sulfonamide lithium salt (Li-TFSI, 99.95%, Sigma-Aldrich, St. Louis, MO, USA) solution (574.2 mg of Li-TFSI in 1 mL of acetonitrile), and 36.2 µL of 4-tert-butylpyridine (98%, Sigma-Aldrich, St. Louis, MO, USA) in 1 mL of chlorobenzene (99.8%, Sigma-Aldrich, St. Louis, MO, USA) at room temperature for 2 h. The spiro-OMeTAD was spin-coated at 4000 rpm for 60 s, followed by drying in a glovebox overnight. The above procedures were implemented inside a dried air-filled glovebox at a dew point of approximately −40 °C. Finally, the Au electrode was deposited by the thermal evaporator.

### 3.5. Characterization of Perovskite Solar Cells

The morphology and thickness were characterized by field emission scanning electron microscope (FE-SEM, Hitachi S4700). The photovoltaic properties of perovskite solar cells were characterized by the solar simulator (Newport Corp., Model 94022A, Irvine CA, USA) at the Future Energy Convergence Core Center (FECC) with a source meter (Keithley Instruments Inc., Keithley 2400, Cleveland, OH, USA) under AM 1.5 illumination (100 mW/cm^2^). The incident photon to electron conversion efficiencies were obtained by the solar cell QE measurement system (PV Measurements Inc., QEX7, Point Roberts, WA, USA). The formation of crystalline TiO_2_ and perovskite was confirmed using an X-ray diffractometer (Rigaku Corp., D/max-2500, Tokyo, Japan). The transmittance measurements were obtained using a UV-vis spectrophotometer (Jasco International Co., Ltd., V-730, Tokyo, Japan). The electrochemical impedance spectroscopy (EIS) was measured at 250 mV applied bias under 1 sun light (1 Hz–300 kHz). The photoluminescence (PL) was measured with an excitation light source at 325 nm and an applied laser power of 50 mW.

## 4. Conclusions

We prepared nanopatterned mesoporous TiO_2_ (mp-TiO_2_) thin-film layers with different aspect ratios (the diameter was 280 nm while the depths were varied: 75 nm, 97 nm, 127 nm, or 167 nm) by using the imprinting method. Perovskite solar cells were then fabricated with the nanopatterned mp-TiO_2_ thin-film layers with different aspect ratios for better energy conversion efficiency (ECE). The ECE of perovskite solar cells without the nanopatterned mp-TiO_2_ thin-film layer is 14.07%. With the nanopatterned mp-TiO_2_ thin-film layer, the ECE of the perovskite solar cells increased from 14.50% (depth: 75 nm) to 15.83% (depth: 127 nm) because the transmittance of the nanopatterned mp-TiO_2_ thin-film layer improved at a wavelength greater than 400 nm that affected the electron generation. Greater electron generation increased the *J_sc_* and electron density on the active layer of perovskite solar cells, which affected *ff*. Moreover, an increase in electron density shifted the Fermi level of TiO_2_ to a more negative potential that reflects to the increment of *V_oc_*. The IPCE spectra also shows similar tendency with the transmittance spectra of the nanopatterned mp-TiO_2_ thin-film layer at a wavelength above 430 nm, which indicates that high transmittance is related to electron generation on perovskite solar cells. However, when the perovskite solar cells were fabricated with a 127 nm depth nanopatterned mp-TiO_2_ thin-film layer, the ECE decreased as the depth of nanopattern went beyond a certain depth. The non-optimized nanopatterned mp-TiO_2_ (75 nm, 97 nm, and 167 nm) shows relatively low efficiency due to the decrease in transmittance. Therefore, in our study, the aspect ratio with a width of 280 nm and a depth of 127 nm was optimized. Our research suggests that the optimized nanopatterned mp-TiO_2_ thin-film layer can be controlled by the imprinting method and can be applied on perovskite solar cells for better ECE with better transmittance. This study expects the optimized nanopatterned mp-TiO_2_ thin-film layer to be implemented in other types of solar cells. New features can be developed using the best aspect ratio, which can be optimized by using machine learning algorithms and AI for a better performance.

## Figures and Tables

**Table 1:** Photovoltaic properties of perovskite solar cells with and without a nanopatterned mp-TiO_2_ thin-film layer.

mp-TiO_2_ Thin-Film Layer		*J_sc_* (mA/cm^2^)	*V_oc_* (V)	*ff*	*η* (%)
(a)	non-nanopatterned	23.30	0.869	0.69	14.07
(b)	nanopatterned to a depth of 75 nm	23.50	0.879	0.70	14.50
(c)	nanopatterned to a depth of 97 nm	24.20	0.893	0.71	15.30
(d)	nanopatterned to a depth of 127 nm	24.62	0.896	0.72	15.83
(e)	nanopatterned to a depth of 167 nm	23.95	0.873	0.68	14.24

## Data Availability

Not applicable.
